# The Role of miRNA-132 against Apoptosis and Oxidative Stress in Heart Failure

**DOI:** 10.1155/2018/3452748

**Published:** 2018-02-25

**Authors:** Xuelei Liu, Zhou Tong, Keyan Chen, Xiaofang Hu, Hongxu Jin, Mingxiao Hou

**Affiliations:** ^1^General Hospital of Shenyang Military Area Command, No. 83 Wenhua Road, Shenyang, Liaoning Province 110016, China; ^2^China Medical University, No. 77 Puhe Road, Shenyang North New Area, Shenyang, Liaoning Province 110122, China

## Abstract

**Objective:**

To explore the effect of microRNA-132 of heart failure and provide theoretical guidance for clinical treatment of heart failure (HF).

**Methods:**

Peripheral blood was collected from HF patients. RT-qPCR was used to determine microRNA-132 expression. Mouse models of heart failure were established. Color Doppler ultrasound was utilized to measure the changes of cardiac function. HE and Masson staining were applied to observe pathological changes of the myocardium. After H_9_C_2_ cells were transfected with microRNA-132, MTT assay was employed to detect the stability of H_9_C_2_ cells. ELISA was used to measure the levels of oxidative stress factors. Western blot assay and RT-qPCR were utilized to determine the expression of Bax, Bcl-2, TGF-*β*1, and smad3.

**Results:**

MicroRNA-132 expression was downregulated in HF patients' blood. After establishing mouse models of HF, cardiac function obviously decreased. HE staining revealed the obvious edema and hypertrophy of cardiomyocytes. Masson staining demonstrated that cardiomyocytes were markedly fibrotic. After microRNA-132 transfection and H_9_C_2_ cell apoptosis induced by H_2_O_2_, antioxidant stress and antiapoptotic ability of the H_9_C_2_ cells obviously increased. TGF-*β*1 and smad3 expression remarkably diminished.

**Conclusion:**

Overexpression of microRNA-132 dramatically increased the antioxidant stress and antiapoptotic ability of H_9_C_2_ cells and decreased the expression of TGF-*β*1 and smad3.

## 1. Background

Heart failure is a complex set of clinical syndromes, which generates cardiac structure or dysfunction leading to ventricular filling or impaired ejection function [1]. A variety of heart diseases, whose clinical manifestations are dyspnea, fatigue, and fluid retention, end up with heart failure. Due to the high morbidity and mortality of heart failure, currently, there is no particularly ideal treatment plan [2].

MicroRNA (miRNA) is endogenous noncoding small RNA containing 18–25 nucleotides and is highly conserved during evolution, regulates the gene expression after transcription through sequence-specific interaction of target genes, and participates in many biological processes [3, 4]. miRNA not only regulates a single gene, but also acts on functionally related gene networks, resulting in complex genetic regulatory networks [5]. Therefore, the study of miRNA is important for studying the occurrence and development of different diseases.

Alzahrani et al. [6] have found that increased expression of miRNA-132 can reduce the incidence of chronic colitis associated tumors. Wang et al. [7] have found that miRNA-132 inhibits the proliferation of human breast cancer cells by directly targeting FOXA1 gene. Nevertheless, the role of miRNA-132 in chronic HF has not been reported yet. The present study investigated the effect of miRNA-132 in HF models* in vivo* and* in vitro* to provide a theoretical basis for targeted therapy of clinical drugs.

## 2. Materials and Methods

### 2.1. Clinical Data

We recruited 67 HF patients, who visited the General Hospital of Shenyang Military in China from January to September 2017, including 30 males and 35 females, at the mean age of 62.17 ± 3.22 years. Of them, 37 patients were in cardiac function of grade III and 28 patients were in cardiac function of grade IV. Inclusion criteria: patients presenting with all of the following criteria were considered for study inclusion: HF was diagnosed in accordance with the Framingham diagnostic criteria combined with echocardiography. Dyspnea appeared after sleep at night, but could gradually ease after sitting. Echocardiography revealed heart chamber expansion and the decreased left ventricular ejection fraction < 45%. Exclusion criteria: patients with one or more of the following conditions were excluded from this study: infectious disease, autoimmune disease, acute cardiovascular and cerebrovascular diseases, rheumatic disease, diabetes, and parathyroid tumor. All patients signed informed consent. Sixty-five patients, who received physical examination from January to September 2017, were recruited as the control group, including 35 males and 27 females, at the mean age of 64.5 ± 7.29 years.

### 2.2. Animals and Cells

Twenty 6-week-old male C57/B6 mice, weighing 23–25 g, were provided by the Experimental Animal Department of China Medical University (production license number SCXK (Liao) 2013-0001, application license number SYXK (Liao) 2013-0007). The project has been approved by the Institutional Animal Care and Use Committee of China Medical University (approval number 2016629). Mice were raised according to international standards. H_9_C_2_ cells were purchased from cell repository of Chinese Academy of Sciences.

### 2.3. Groups

Mice were randomly divided into two groups: the sham surgery group (sham group) and HF group. H_9_C_2_ cells were divided into three groups: NC group, miRNA-132 mimics group, and miRNA-132 inhibitor group.

### 2.4. Sample Collection

Four weeks after establishing HF model, all mice were sacrificed by bloodletting under the anesthesia. Mouse venous blood was collected, and serum was used for ELISA. Mouse heart tissue was harvested. One portion of heart tissue was preserved in the liquid nitrogen for quantitative real-time PCR. The remainder was fixed in formaldehyde for HE staining and Masson staining.

### 2.5. Establishment of a HF Model

HF model was established as previously reported with minor modifications [8]. After intraperitoneal anesthesia with 2% sodium pentobarbital, mice were connected to the small animal respirator, anesthesia machine, and monitor. An incision was made 0.5 cm above the manubrium sterni to find the aortic arch of mice. After the 27 G pinhead was ligated with the aortic arch, the pinhead was pulled out, and the skin was closed.

### 2.6. Color Doppler Ultrasound

Four weeks after surgery, mice were weighed. After anesthesia, the mice were fixed in the supine position, and the chest was shaved. Echocardiography (Philips CX50; probe model: S-12-4; frequency: 4–12 MHz) was used to measure left ventricular end-diastolic diameter (LVIDd), left ventricular end-systolic diameter (LVIDs), and ejection fraction (EF). The average value of three cardiac cycles was calculated.

### 2.7. HE Staining

The myocardium was fixed in formaldehyde for 48 hours, dehydrated, permeabilized, and embedded in paraffin. Paraffin tissue was sliced into 0.4 *μ*m serial sections. These sections were deparaffinized, washed with PBS, stained with hematoxylin for 3 minutes, differentiated in a hydrochloric acid ethanol mixture for 3–5 minutes, and then stained with eosin for 1 minute. The sections were dehydrated through a graded alcohol series and permeabilized. The pathological changes in the myocardium were observed under the microscope.

### 2.8. Masson Staining

Paraffin sections were dewaxed, hydrated, and treated with distilled water, followed by Regaud dye hematoxylin staining with Masson for 5–10 min. After being fully washed, sections were treated with Ponceau Fuchsin Acid Solution for 5–10 min, immersed in 2% acetic acid aqueous solution for a moment, and differentiated in 1% phosphomolybdic acid aqueous solution for 3–5 min. Without washing with water, these sections were treated with aniline blue for 5 min, immersed in 0.2% acetic acid aqueous solution for a moment, in 95% alcohol, anhydrous alcohol, permeabilized with xylene, and mounted with neutral resin.

### 2.9. ELISA

After serum isolation, serum BNP levels and SOD (SES134Mu) and MDA (CEA597Ge) levels in the supernatant were determined in strict accordance with the instruction of the kit. Optical density (OD) values were measured at 450 nm with the microplate reader. The standard curves of OD value and concentration were drawn, and the concentration of the sample was calculated according to the standard curve.

### 2.10. Transfection

miRNA-132 mimics and miRNA-132 inhibitor lentiviral vectors (GenePharma, Shanghai, China) were constructed. Cells at 1 × 10^5^ were incubated in 6-well plates, and lentiviral transfection solution at Multiply of Infection (MOI) = 50 was added. Simultaneously, polybrene at a final concentration of 5 *μ*g/mL was added. 8–12 hours later, cell morphology was observed. 72–96 hours later, cells were harvested. Real-time fluorescence quantitative PCR was used to determine miRNA-132 transfection.

### 2.11. MTT Assay

Empty vector-transfected H_9_C_2_ cells at 8000 cells/well were paved in the 96-well plate, incubated with 100, 200, 400, 600, and 800 *μ*mol/L H_2_O_2_ at 37°C and 5% CO_2_ for 24 hours. 20 *μ*l of MTT (Sigma, USA) was added to each well for 4 hours. After removal of the medium, 150 *μ*l of dimethyl sulfoxide was added in each well (Sigma) and shaken at a low speed for 10 minutes. OD values of each well were measured at 490 nm with a microplate reader. Cell viability was calculated as follows: (OD_sample_ − OD_sample  blank_)/(OD_control_ − OD_control  blank_) × 100%.

### 2.12. Real-Time Fluorescence Quantitative PCR

Primers were designed according to the miRNA-132, Bax, Bcl-2, TGF-*β*, and smad3 sequences reported in GenBank and synthesized by Invitrogen Company. Total RNA was extracted from myocardium and H_9_C_2_ cells. First-strand DNA was synthesized by reverse transcription. Real-time fluorescence quantitative PCR was performed according to real-time fluorescence quantitative PCR kit. PCR amplification was conducted as follows: 95°C 30 s, 95°C 5 s, and 60°C 30 s; collecting the fluorescence signal; 40 cycles. The relative gene expression was analyzed with the 2^∧^(−Delta Delta CT) method. The primers used for real-time PCR are as follows: miRNA-132 human, forward: 5′-CGATTGTTACTGTGGGAA-3′; miRNA-132 mice, forward: 5′-ACAGTCTACAGCCATGGT-3′; Bax, forward: 5′-TGGCAATGTTGAGCTCCAAA-3′; reverse: 5′-GCAGGGTGGTGGCACTGT-3′; Bcl-2, forward: 5′-TGATAACCGGGAGATCGTGA-3′; reverse: 5′-GGAGATGAAGACTCCGCGC-3′; TGF-*β*1, forward: 5′-CTGATTACCATGAGTTGG-3′; reverse: 5′-CATATGGAGATCATGGGC-3′; smad3, forward: 5′-CTGCTCTCCAATGTCAACAG-3′; reverse: 5′-TGACAGCGCCATCTTTGTC-3′; GAPDH, forward: 5′-CTTGTGCTGTAAGATCGAG-3′; reverse: 5′-CATACTTATCTCCTTGTA-3′

### 2.13. Western Blot Assay

Total protein was extracted from H_9_C_2_ cells, which was quantified with BCA Protein Assay Kit and subjected to SDS-PAGE, transferred onto the membranes. The membranes were blocked with 5% defatted milk powder and incubated with Bax, Bcl-2, TGF-*β*, and p-smad3 antibodies at 4°C overnight and then with horseradish peroxidase-labeled secondary antibody at room temperature for 2 hours. Proteins were visualized with enhanced chemiluminescence kit and gel imaging system. Results were analyzed by Image Tools.

### 2.14. Statistical Analysis

Data were analyzed using SPSS 19.0 statistical software; measurement data were expressed as mean ± standard deviation. Data between groups were compared using independent samples* t*-test. Data among groups were compared using one-way analysis of variance (ANOVA) with S-N-K test analysis. A value of *P* < 0.05 was regarded as a significant difference.

## 3. Results

### 3.1. Downregulation of miRNA-132 Expression in Peripheral Blood of HF Patients

**Figure 1:**
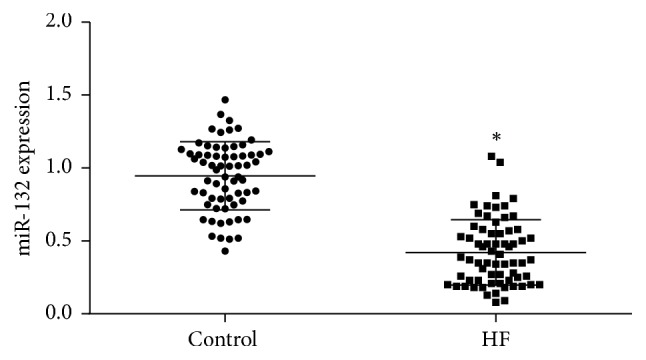
Real-time fluorescence quantitative PCR for detecting miRNA-132 expression after collecting peripheral blood, extracting total RNA, and synthesis of the first-strand DNA by reverse transcription. ^*∗*^*P* < 0.05, versus control group.

Real-time fluorescence quantitative PCR results showed that miRNA-132 expression significantly diminished in peripheral blood of HF patients compared with the control group (Figure 1).

### 3.2. Establishment of Mouse Models of HF

**Figure 2:**
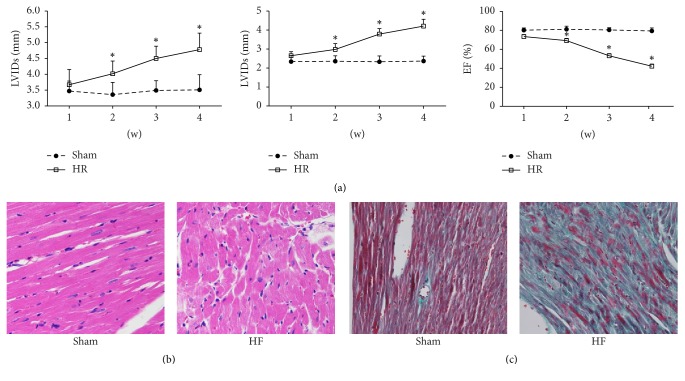
Following the establishment of mouse models of HF, (a) changes in cardiac function in mice measured by echocardiography, (b) pathological changes of the myocardium in mice observed by HE staining, and (c) changes of myocardial fibrosis in mice observed by Masson staining. ^*∗*^*P* < 0.05, versus control group.

Color Doppler ultrasound results demonstrated that, 1 week after model establishment, LVIDs and LVIDd increased, but EF did not significantly alter compared with the sham group (*P* > 0.05) (Figure 2(a)). At 2-3 weeks, LVIDs and LVIDd persistently increased, but EF persistently reduced. At 4 weeks, LVIDs and LVIDd significantly increased (*P* < 0.05 versus sham group), but EF significantly decreased (*P* < 0.05 versus sham group). HE staining (Figure 2(b)) results exhibited that HF group showed obvious edema, hypertrophy, and cell swelling compared with sham group. Masson staining (Figure 2(c)) results displayed that cardiomyocytes in the HR group had obvious fibrotic changes compared with the sham group. These results suggest that aortic arch coarctation indicated successful establishment of a HF mouse model.

### 3.3. Downregulation of miRNA-132 Expression in a Mouse Model of HF

**Figure 3:**
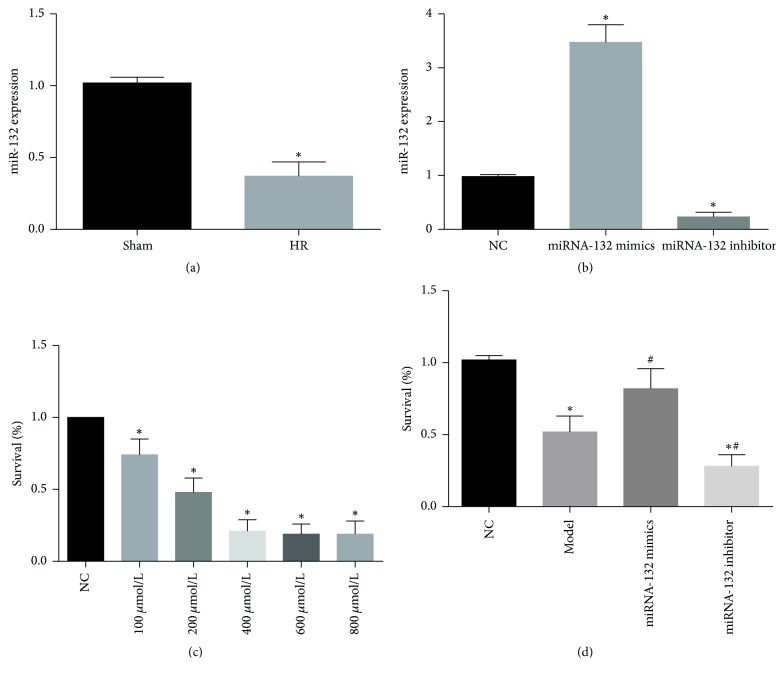
(a) miRNA-132 expression detected by real-time fluorescence quantitative PCR after extracting total RNA from mouse myocardium; (b) miRNA-132 expression detected by real-time fluorescence quantitative PCR after transfection of H_9_C_2_ cells with NC, miRNA-132 mimics, and miRNA-132 inhibitor; (c) H_2_O_2_ induced H_9_C_2_ cells to simulate HF model* in vitro*; (d) stability of H9C2 cells transfected with NC, miRNA-132 mimics, and miRNA-132 inhibitor measured by MTT assay. ^*∗*^*P* < 0.05, versus sham group; ^*∗*^*P* < 0.05, versus NC group; ^#^*P* < 0.05, versus model group.

To investigate the effect of miRNA-132 on HF, real-time fluorescence quantitative PCR was used to determine miRNA-132 expression in mouse myocardium. Data suggested that miRNA-132 expression significantly reduced in HF group compared with the sham group (*P* < 0.05) (Figure 3(a)). Real-time fluorescence quantitative PCR results confirmed that miRNA-132 plays a negative regulatory role in HF.

### 3.4. miRNA-132 Overexpression Enhances the Stability of H_9_C_2_ Cells

To further verify the effect of miRNA-132 in mouse models, we selected H_9_C_2_ cell line. miRNA-132 expression was detected with real-time fluorescence quantitative PCR after transfection with NC, miRNA-132 mimics, and miRNA-132 inhibitor (Figure 3(b)). Data suggested that miRNA-132 expression significantly increased after transfection with miRNA-132 mimics compared with the NC group (*P* < 0.05). miRNA-132 expression significantly diminished after transfection with miRNA-132 inhibitor compared with the NC group (*P* < 0.05). H_9_C_2_ cells were induced by H_2_O_2_* in vitro* to simulate HF. Data suggested that when H_2_O_2_ concentration reached 200 *μ*mol/L, the survival rate of NC-transfected H_9_C_2_ was 0.46 ± 0.12%  (Figure 3(c)). We selected 200 *μ*mol/L as a concentration to simulate heart failure* in vitro*. We further determined the stability of cells transfected with miRNA-132. Data indicated that cell stability significantly increased after transfection with miRNA-132 mimics compared with the NC group (*P* < 0.05). However, cell stability significantly reduced after transfection with miRNA-132 inhibitor compared with the NC group (*P* < 0.05; Figure 3(d)). These findings indicated that overexpression of miRNA-132 increased the stability of H_9_C_2_ cells.

### 3.5. Overexpression of miRNA-132 Suppresses Oxidative Stress of H_9_C_2_ Cells

**Figure 4:**
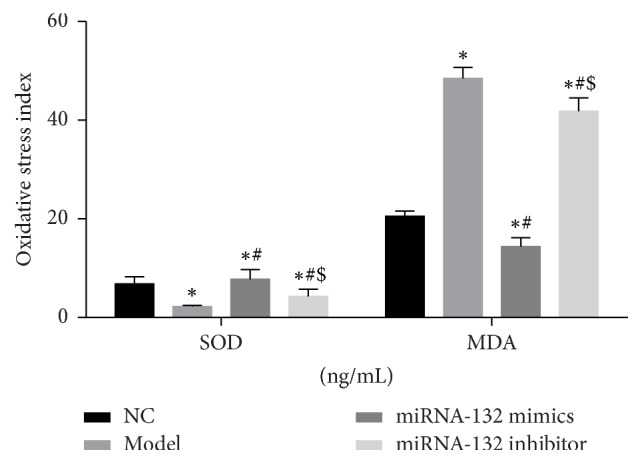
SOD and MDA levels in the supernatant of H_9_C_2_ cell culture as determined by ELISA. ^*∗*^*P* < 0.05, versus sham group; ^*∗*^*P* < 0.05, versus NC group; ^#^*P* < 0.05, versus model group; ^$^*P* < 0.05, versus miRNA-132 mimics.

To verify miRNA-132 function, ELISA was utilized to detect SOD and MDA levels in the supernatant. As exhibited in Figure 4, SOD expression significantly decreased (*P* < 0.05), but MDA expression significantly increased (*P* < 0.05) in the model group compared with the NC group. After overexpression of miRNA-132, SOD expression significantly increased (*P* < 0.05), but MDA expression significantly reduced (*P* < 0.05) compared with the model group. Nevertheless, after inhibiting miRNA-132 expression, SOD expression significantly diminished (*P* < 0.05), but MDA expression significantly increased (*P* < 0.05) compared with the miRNA-132 mimics group. These findings suggested that overexpression of miRNA-132 could obviously inhibit oxidative stress induced by H_2_O_2_ in H_9_C_2_ cells.

### 3.6. Overexpression of miRNA-132 Mitigates H_9_C_2_ Cell Apoptosis

**Figure 5:**
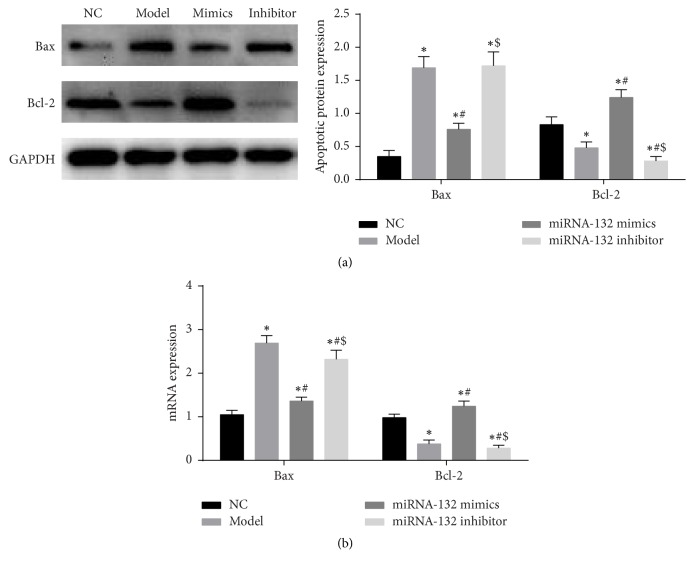
(a) Bax and Bcl-2 expression in H_9_C_2_ cells as detected by western blot assay; (b) Bax and Bcl-2 mRNA expression as measured by RT-qPCR. ^*∗*^*P* < 0.05, versus sham group; ^*∗*^*P* < 0.05, versus NC group; ^#^*P* < 0.05, versus model group; ^$^*P* < 0.05, versus miRNA-132 mimics.

Western blot assay and real-time fluorescence quantitative PCR were used to measure apoptosis related factors Bax and Bcl-2 expression. Western blot assay results (Figure 5(a)) showed that Bax expression significantly increased (*P* < 0.05), but Bcl-2 expression significantly reduced (*P* < 0.05) in the model group compared with the NC group. After overexpression of miRNA-132, Bax expression significantly diminished (*P* < 0.05), but Bcl-2 expression significantly increased (*P* < 0.05) compared with the model group. After inhibiting miRNA-132 expression, Bax expression significantly increased (*P* < 0.05), but Bcl-2 expression significantly reduced (*P* < 0.05) compared with the miRNA-132 mimics group. Real-time fluorescence quantitative PCR results were consistent with that of western blot assay (Figure 5(b)). These findings suggested that overexpression of miRNA-132 could dramatically suppress H_9_C_2_ cell apoptosis induced by H_2_O_2_.

### 3.7. Overexpression of miRNA-132 Lessens the Expression of TGF-*β*1 and smad3

**Figure 6:**
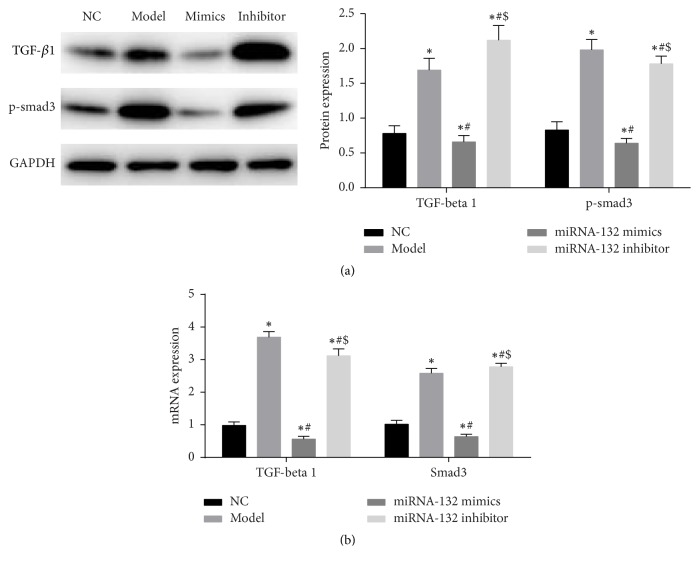
(a) TGF-*β*1 and smad3 protein expression in H_9_C_2_ cells as measured by western blot assay; (b) TGF-*β*1 and smad3 mRNA expression in H_9_C_2_ cells as determined by RT-qPCR. ^*∗*^*P* < 0.05, versus sham group; ^*∗*^*P* < 0.05, versus NC group; ^#^*P* < 0.05, versus model group; ^$^*P* < 0.05, versus miRNA-132 mimics.

Western blot assay and real-time fluorescence quantitative PCR were utilized to measure the expression of TGF-*β*1 and p-smad3. Western blot assay results (Figure 6(a)) revealed that TGF-*β*1 and p-smad3 expression significantly increased in the model group compared with the NC group (*P* < 0.05). After overexpression of miRNA-132, TGF-*β*1 and p-smad3 expression significantly decreased compared with the model group (*P* < 0.05). After suppressing miRNA-132 expression, TGF-*β*1 and p-smad3 expression significantly increased compared with the miRNA-132 mimics group (*P* < 0.05). Real-time fluorescence quantitative PCR results were consistent with that of western blot assay (Figure 6(b)).

## 4. Discussion

This study established a mouse model of HF to explore miRNA-132 effect.* In vitro* experiments simulated the occurrence of HF to further explore miRNA-132 effect, which provides the theoretical basis for the research and development of HF drugs.

Cardiomyocyte apoptosis, ventricular remodeling, and myocardial fibrosis are important pathophysiological processes of cardiomyocyte repair and overall compensation after HF [9–11]. Ventricular remodeling and myocardial fibrosis after HF seriously affect the quality of life of patients. In this study, mouse models of chronic HF were established by coarctation of the aortic arch in mice, which greatly shortened the time required to establish a HF model by narrowing the abdominal aorta. Color Doppler ultrasound revealed that LVIDs and LVIDd remarkably increased, but EF obviously reduced. HE staining showed pathological changes, including swelling and hypertrophy of cardiomyocytes. Masson staining demonstrated obvious fibrosis of cardiomyocytes. These findings confirmed that the establishment of a mouse model of coarctation of the aorta greatly shortened the time required to establish a model, which provides a new method for the establishment of animal models of HF.

miRNA is a class of noncoding RNA with regulatory function found in eukaryotes in recent years. miRNA is mainly involved in the regulation of posttranscriptional genes [12]. After maturation, miRNA can regulate gene expression by complementation with target mRNA, thereby regulating cell differentiation, growth, proliferation, metabolism, and apoptosis [13–16]. The study of miRNA-132 in pancreatic cancer is adequate. Previous studies showed that miRNA-132 plays a role in inhibiting cell proliferation by acting on the retinal tumor suppressor gene Rb [17, 18]. In this study, peripheral blood of HF patients was collected for real-time fluorescence quantitative PCR. Our results demonstrated that miRNA-132 expression obviously reduced in peripheral blood. The mouse HF model was established, and the expression of miRNA-132 was detected by PCR. The results were consistent with the clinical results. We assumed that miRNA-132 may play a negative regulatory role in the occurrence and development of HF. To further verify the effect of miRNA-132, we simulated HF models* in vitro*. H_9_C_2_ cells were transfected with miRNA-132 mimics and miRNA-132 inhibitor. After overexpression of miRNA-132, H_9_C_2_ cells could obviously resist oxidative stress and apoptosis induced by H_2_O_2_.

TGF-*β*1, as an important profibrosis factor, has a significant role in promoting fibrosis [19, 20]. TGF-*β*1 plays multiple regulatory roles in fibrosis remodeling through smad's dependent pathway in many diseases [21]. During HF or myocardial ischemia, TGF-*β*1 signaling can induce fibrosis of cardiac fibroblasts and promote the synthesis of collagen and fibronectin, finally promoting myocardial fibrosis [22]. In the present study, H_9_C_2_ cells were transfected with miRNA-132 mimics and miRNA-132 inhibitor. We found that, after overexpression of miRNA-132, TGF-*β*1 and smad3 expression noticeably diminished. We assumed that miRNA-132 may play a role in inhibiting cardiomyocyte fibrosis through TGF-*β*1 signaling pathway, but the precise mechanism requires further investigations.

This study revealed that overexpression of miRNA-132 has antioxidative stress and antiapoptotic effects, laying a theoretical foundation for the research and development of clinical targeted drugs. Nevertheless, to conclude if miRNA-132 is an antioxidant and antiapoptotic or whether it is an anticardiomyocyte fibrosis that acts through the TGF-*β*1 signaling pathway deserves our further investigations.
